# Changes in peripheral blood mononuclear cell electrical properties in response to viral exposure and vaccination

**DOI:** 10.1038/s41598-025-08724-6

**Published:** 2025-07-09

**Authors:** Krista S. P. Clarke, Alexander T. Stewart, Emma L. Sinclair, Rebecca Lewis, Fatima H. Labeed, Deborah K. Dunn-Walters, Michael Pycraft Hughes

**Affiliations:** 1 Department of Biomedical Engineering and Biotechnology, Khalifa University of Science and Technology, Abu Dhabi, UAE; 2 Centre for Biomedical Engineering, School of Mechanical Engineering Sciences, University of Surrey, Guildford, Surrey GU2 7XH UK; 3 School of Bioscience and Medicine, University of Surrey, Guildford, Surrey GU2 7XH UK; 4 Department of Comparative Biomedical Sciences, School of Veterinary Medicine, University of Surrey, Guildford, Surrey GU2 7XH UK; 5 Department of Biology, United Arab Emirates University, Al Ain, UAE

**Keywords:** SARS-CoV-2, Correlate of protection, Dielectrophoresis, Peripheral blood mononuclear cell, Membrane conductance, Membrane capacitance, Cytoplasmic conductivity, Biomarkers, Membrane biophysics, Viral infection

## Abstract

Viral infection triggers both cellular and systemic responses in vivo. These are sometimes difficult to study since the immune system is subject to constant challenge from multiple pathogens. However, during the COVID-19 pandemic, a unique opportunity arose to measure the body’s immune response during lockdown, when exposure to pathogens from the environment was substantially lower. This allowed measurement of COVID-naïve patients, as well as those who had recovered from COVID-19. The effects of subsequent vaccination and boosters could also be assessed. This offers two advantages; an insight into the humoral response to novel pathogens, and the opportunity to measure the ability of the body to respond to challenge from both new pathogens and those to which exposure had already occurred. In this paper, we used dielectrophoresis (DEP) to analyze the electrophysiological fingerprint of peripheral blood mononuclear cells (PBMCs) from donors who had never had COVID-19, those who had recovered from COVID-19, and those who received first, second, or third vaccine doses. This was performed before and after incubation with the receptor binding domain of the SARS-CoV-2 spike protein to determine whether differential changes in the electrical properties of PBMCs could be detected and evaluated. Clear trends in response over time were observed, suggesting that DEP could pave the way towards a new correlate of protection (CoP) to SARS-CoV-2. Furthermore, since the test measures immune response to challenge, it may be widely applicable to other diseases.

**Supplementary Information:**

The online version contains supplementary material available at 10.1038/s41598-025-08724-6.

## Introduction

The humoral immune response involves complex interactions of multiple cell types to recognize invading cells through identification and subsequent chemical memory of surface molecules on the target organism. The process of responding to both primary infection and reinfection is complex, and difficult to study in vivo in humans; we are all exposed to countless viruses through the day, most commonly through respiratory tract infections acquired from everyday interaction with others. Consequently, our immune response is almost always in a state of active readiness, rather than retreating to an unchallenged, quiescent state. An almost unique change from this state occurred during 2020–21, when the Coronavirus disease-2019 (COVID-19) pandemic caused substantial portions of the population to be quarantined at home. This reduction in person-person interaction prevented the spread of COVID-19, but also led to a substantial reduction in all other infections^1–4^. This allowed study, for a brief period, of the immune response of those previously unaffected by COVID-19, those who had been vaccinated or had recovered, in the unusual scenario of a population whose immune response had been largely unchallenged in the preceding months.

COVID-19 is a severe pneumonia-associated respiratory syndrome caused by the novel beta coronavirus strain, severe acute respiratory syndrome coronavirus-2 (SARS-CoV-2)^5,6^. From the first outbreak reported in December 2019 in Wuhan, China, SARS-CoV-2 quickly spread and became prevalent worldwide, globally threatening public health, global economies, increased poverty, and social cohesion. The World Health Organization (WHO) declared it a global pandemic on March 11, 2020^6–9^. By April 2021, 219 countries and territories had reported cases of COVID-19, and there was a global death toll of 3,076,021^10^. In August 2022, the global death toll had increased to more than 6 million^9^. Previous infection with SARS-CoV-2 has been shown to provide protection against reinfection in rhesus macaques^11,12^, and both BNT162b2 and ChAdOx1-S vaccines have been shown to be highly effective at reducing the risk of hospitalization, severe disease, and death due to COVID-19^13–16^. However, there is also substantial evidence that immune protection against COVID-19 declines over time. The number of antibodies^17–23^, antibody neutralizing activity, and T-cell immune response^24^ tends to decrease over time, and the risk of COVID-19 reinfection increases^25^. From the start of the pandemic, newspaper headlines vocalized the questions of the world’s population, such as “How long does COVID immunity really last?”—The Telegraph, “COVID immunity: Can you catch it twice?”—BBC News, and “COVID reinfections in the UK: how likely are you to catch coronavirus again?”—The Guardian^26–28^.

Strong evidence shows asymptomatic, mild, or severe SARS-COV-2 infection induces robust CD4^+^ T-cell and CD8^+^ T-cell responses^29–33^. Studies have shown 93% of convalescent patients possess detectable circulating CD4^+^ T cells specific to SARS-CoV-2 S-protein, N-protein, and M-protein^29,34,35^, and 50% of acute COVID-19 cases^35^. Virus specific follicular T-cells, required for IgG and high quality neutralizing antibody (NAb) responses, make up a significant proportion of SARS-CoV-2 specific CD4^+^ cells in acute and convalescent COVID-19^35^. In addition to serum antibodies, memory T and B cell responses are important in the protection of SARS-CoV-2 infection and modulate SARS-CoV-2 severity^29,36–38^. Memory T-cell responses have been shown to occur following mild infection, as evident by the formation of T-dependent IgG RBD-specific memory B-cells^33^. Memory T-cell responses are present within a few weeks post-symptom onset (PSO)^39^, with SARS-CoV-2 specific CD4^+^ T-cells detected as early as day four PSO^35^ and are still present in more than 90% of subjects more than 6 months PSO^34,40^. Robust CD8^+^ T-cell responses also occur, and 70% of recovered COVID-19 patients establish CD8^+^ memory responses^29^ within 1-month PSO, decreasing to 50% 6–8 months PSO^34^. Longitudinal data published by Gangaev et al.^41^ supports this, reporting SARS-CoV-2 specific CD8^+^ T-cell responses in severe COVID patients remain 5 months following discharge from hospital, and transition to functional memory T-cells^41^.

Immune markers which statistically correlate with a level of protection that prevents a clinically relevant outcome are known as correlates of protection (CoP)^42–44^. Identifying easily testable CoP for viruses such as SARS-CoV-2 allow assessment of risk of symptomatic disease and requirements for vaccination/revaccination, determination of population-level immunity, informing immunization programs, and developing recommendations on public health and social measures^43,45^. A validated CoP would also prove invaluable in the approval and clinical validation of new SARS-CoV-2 vaccines, assessing efficacy of existing vaccines in new populations and against other variants of concern (VOC), in addition to evaluating vaccine regimen modifications^46^. Although most research into CoP focus on antibody-mediated protection, growing evidence demonstrates that cellular immune responses provide fundamental protection from SARS-CoV-2, and clearance of SARS-CoV-2 infection relies on CD4^+^ and CD8^+^ T-cell responses as well as NAb action^37^. To address this, it is necessary to identify alternative CoP that can be measured quickly and at low cost.

In this paper, we examine the electrical properties of PBMCs using dielectrophoresis (DEP), which measures the passive electrical properties of cells^47^. DEP uses the interaction between cells and multifrequency electric fields to determine the mean membrane conductance and capacitance of cell ensembles of typically tens of thousands of cells. Whilst DEP is most commonly used for analysis of single cell types, it has been shown to be effective in identifying oral cancer^48,49^ and bladder cancer^50^ through the analysis of heterogeneous biological samples acquired from brush biopsy or urine sample. Given the changes in the electrical properties of T-cells and B-cells following T-cell receptor (TCR) and B-cell receptor (BCR) activation^51,52^, such as increased intracellular Ca^2+^ ion concentrations, we hypothesized that the electrical response of PBMCs to stimulation with SARS-COV-2 is different in individuals who possess immune memory. As DEP is a fast, low-cost, and easily adoptable technique, it was investigated whether differences in the electrical properties of PBMCs following stimulation with SARS-CoV-2 specific antigens could pave the way towards a new CoP to SARS-CoV-2.

## Materials and methods

### Donors

A favorable ethical opinion was given to this study by the University of Surrey Ethics Committee (UEC/2017/052/FHMS, FHMS20/21 192 EGA). Changes in the electrical properties of PBMCs following incubation with the SARS-CoV-2 spike protein were investigated in a total of n = 19 different donors. Participants were categorized into four donor cohorts, including COVID-19 Naïve, Recovered COVID-19, Second Dose and Third Dose donors. COVID-19 Naïve donors had not experienced symptoms to suspect SARS-CoV-2 infection since the outbreak of the pandemic and had a negative rapid antibody test (SureScreen Diagnostics COVID-19 Antibody Rapid Test) for IgM and IgG antibodies on the day prior to blood donation. Recovered COVID-19 donors were participants who had previously had COVID-19, confirmed by a positive PCR test, with symptoms lasting 3–4 weeks. All had negative lateral flow tests for COVID-19 on the day prior to blood donation and were not experiencing symptoms of long COVID., Second Dose participants (Vaccinated donors) had received the second dose of a Pfizer/BioNTech or AstraZeneca vaccine and Third Dose participants (Boosted donors) had received the third dose of the Pfizer vaccine.—Of these 19 donors, two donated multiple times in different donor cohorts; one donor donated in the COVID-19 Naïve and boosted donor cohorts, and one donated to the vaccinated and boosted donor cohorts. Two vaccinated donors donated twice at different times after their vaccination (these data are presented in the Supplementary Information). Characteristics of participants recruited for each experiment including age, gender, time between natural infection/vaccination and experiment, and which COVID-19 vaccine they received is outlined in Table 1.

**Table 1:** Baseline characteristics of COVID-19 naïve (n = 5), Recovered COVID-19 (n = 4), Vaccinated (n = 5), and Boosted (n = 7) donor cohorts whose PBMCs were isolated and stimulated for 3 h with the RBD of the COVID-19 spike protein.

	COVID-19 Naïve (n = 5)	Recovered COVID-19 (n = 4)	Vaccinated (n = 5)	Boosted (n = 7)
Age (years)	37 (± 7)	28 (± 1)	31 (± 14)	29 (± 8)
Gender	Female (3/5)	Male (3/4)	Female (4/5)	Female (4/7)
Ethnicity	White (5/5)	White (4/4)	White (4/5)	White (6/7)
Time since symptom onset (days)	–	58 (± 12)	–	–
Time since latest vaccination (days)	–	–	38 (± 21)	65 (± 20)

Continuous variables are expressed as mean (± SD) and categorical variables as frequency (fractions of the donor cohort).

### Isolation of PBMCs from blood

Peripheral whole blood was collected from all donors by a qualified phlebotomist via venipuncture using BD-Bioscience lithium heparin vacutainers. Complete media RPMI-1640 medium with L-glutamine (ThermoFisher, UK) supplemented with 10% fetal calf serum (FCS; Merck, UK) and 1% penicillin–streptomycin (Merck, UK; 50,000 U/mL penicillin, 50 mg/mL streptomycin solution) was mixed with freshly collected whole blood 1:1. The diluted blood sample was carefully layered on top of Ficoll-paque (GE-healthcare) and centrifuged 400 g, 30 min to isolate PBMCs from the whole blood via standard density centrifugation. A pipette was used to remove and aliquot the buffy layer into falcon tubes, which were topped up with complete media and spun at 400g, 15 min, acceleration/deceleration = 8. The supernatant was discarded, resuspended in complete media, and 10 μL removed for cell counting and radius measurement using the C-Chip Disposable Haemocytometer, DHC-N01 (Labtech, Uckfield, UK) and ImageJ (National Institutes of Health, V1.54d). PBMC concentration was subsequently amended to 10 × 10^6^ cells/ mL suspended in complete media, and 100 μL containing 1 million cells was aliquoted into the required number of wells in a 96-well plate for PBMC stimulation.

### PBMC stimulation

#### Control incubation

PBS (Merck, UK) was added as a vehicle control, after which the cells were incubated at 37 °C, 5% CO_2_ for 3 h.

#### SARS-CoV-2 spike glycoprotein receptor binding domain

PBMCs were stimulated with 500 ng/ mL recombinant human coronavirus SARS-CoV-2 Spike Glycoprotein RBD (Active) (Abcam, ab273065) in complete media at 37 °C, 5% CO_2_ for 3 h. The RBD antigen was chosen as the stimulant as it is responsible for binding to the host cell ACE2 receptors and is highly immunogenic—donors vaccinated with BNT162b1 possess strong CD4^+^ and CD8^+^ T-cell responses to peptides encoding the SARS-CoV-2 RBD^53^, T-dependent IgG RBD-specific memory B-cells are generated following mild infection^33^, 90% of serum antibodies target the RBD^54^ and the RBD is the main target of NAb^55^, with NAb titers to the RBD significantly correlating with neutralization of the SARS-CoV-2 virus^56–58^. The SARS-CoV-2 Spike Glycoprotein RBD (Active) (Abcam, ab273065) chosen to stimulate the PBMCs has been used in previous publications^59–61^.

### Sample preparation

Following stimulation, cell suspensions were centrifuged at 400 g for 5 min, and resuspended in 1 mL DEP medium comprising deionized water diluted with 8.5% (w/v) sucrose, 0.5% (w/v) dextrose, 250 µM MgCl_2_, and 100 µM CaCl_2_, then supplemented with PBS to a final conductivity of 102 mS/m. The cell suspension was spun again at 400 g for 5 min, and the cell pellet resuspended in DEP medium at a volume required for a cell concentration of 1 × 10^6^ cells/ mL.

### DEP analysis

The electrophysiological parameters of PBMCs were subsequently measured using a DEPtech 3DEP (DEParator, UK)^62^ using a 1kHz-45MHz frequency range. Cells were analyzed for 30 s using bands 4–9. A minimum of three technical repeats were measured for each sample. Raw data collected by the 3DEP was analyzed using the 3DEP Data method outlined elsewhere^62^ to obtain values of G_eff_, C_eff_ and σ_cyto_ from DEP spectra modelled with an R squared value^63^ of above 0.8. Mean Difference Values (MDV) were also calculated, by which the average relative DEP force of the highest nine frequencies was subtracted from the average relative DEP force of the lowest nine frequencies^50^.

### Single cell RNA sequencing (scRNAseq)

In parallel with the 3DEP experiments, changes in the gene expression of PBMCs donated from three second dose donors at a single-cell level following 3-h RBD-stimulation using the Cell Ranger (10 × Genomics) were performed. Blood was obtained from three healthy control participants at least 3 weeks after their second COVID-19 vaccination, PBMCs were then obtained by Ficoll-Paque density centrifugation. PBMCs were plated in 96 well plates at 1 × 10^6^ cells/mL in complete media (RPMI, 5% FCS, Pen/strep) supplemented with the following stimuli to 200 µL. For each participant there was one control well (+PBS), one RBD (500ng RBD—Biolegend), and one peptide mix (500ng of Spike, Membrane and Nucleocapsid—Miltenyi PepTivator). Samples were incubated for 3 h at 37 °C, at 2.5h 1 µL of hashtag antibodies (Total-Seq™-C, Biolegend) were added to each well to enable 10X genomics lane multiplexing on a per patient basis. Cells were collected and cooled to 4 °C and washed, 400g at 4 °C, in PBS + 5% FCS and again in PBS + 0.004% non-acetylated BSA. Samples were counted and pooled at equal numbers, washed again and resuspended in PBS + 0.004% non-acetylated BSA to 1,000 cell/uL for single cell transcriptomics. Samples were run on the 10X genomics platform using Cell 5′ Library Kit v1.1, Chromium Single Cell 5′ Gel Bead Kit v1.1 and Chromium Single Cell 5′ Library Construction Kit v1.1 (10 × Genomics) following manufacturer’s protocol for Chromium Single Cell V(D)J Reagent Kits v1.1. Samples were run on an Illumina Nextseq to a read depth of ~ 30,000 reads per cell and fastqs processed in CellRanger. Matrices were read into Seurat V4 and data processing carried out using default filtering, with the additional removal of HLA, IGH and IGL from variable features^64^, with SCTransform used to normalize data, and Harmony was used to converge patient data. Cell IDs were inferred from Stewart et al.^64^) utilizing Seurat’s integration feature. Differential gene expression analysis was carried out using default setting and Gene Set Enrichment Analysis performed using the BTMplus reference (See here for references https://github.com/shuzhao-li/BTM).

### Data analysis and statistical analysis

All statistical analysis was conducted in Prism 9 for Windows (GraphPad Software, San Diego). The D’Agostino & Pearson (“omnibus K2”) test for normality (*p* > 0.05) was used to assess whether baseline values of G_eff_, C_eff_, σ_cyto_, and cell radius were normally distributed, because it a versatile and powerful test which computes how far from Gaussian distribution values are based on skewness and kurtosis. For non-normally distributed data, the non-parametric Kruskal–Wallis test followed by Dunn’s multiple comparisons test was used. For normally distributed data, a one-way ANOVA test followed by Tukey’s multiple comparisons were used to assess the differences in the baseline electrical properties between donor cohorts. Simple linear regression models fitted using least squares regression, were used to assess the trend in the baseline electrical properties of PBMCs against duration of time between the date of the experiment and the date of symptom onset or date of vaccination. The coefficient of the slope, the 95% confidence intervals, and the p-value against the null hypothesis that the overall slope is equal to zero (* < 0.05, ** < 0.01) were considered. The r^2^ value of the regression model was used to determine the variance in G_eff_, C_eff_, σ_cyto_ and cell radius attributable to the number of days between the date of the experiment and the date of COVID-exposure.

## Results

### Exposure to COVID-19 significantly alters the cytoplasm conductivities of PBMCs

**Fig. 1:**
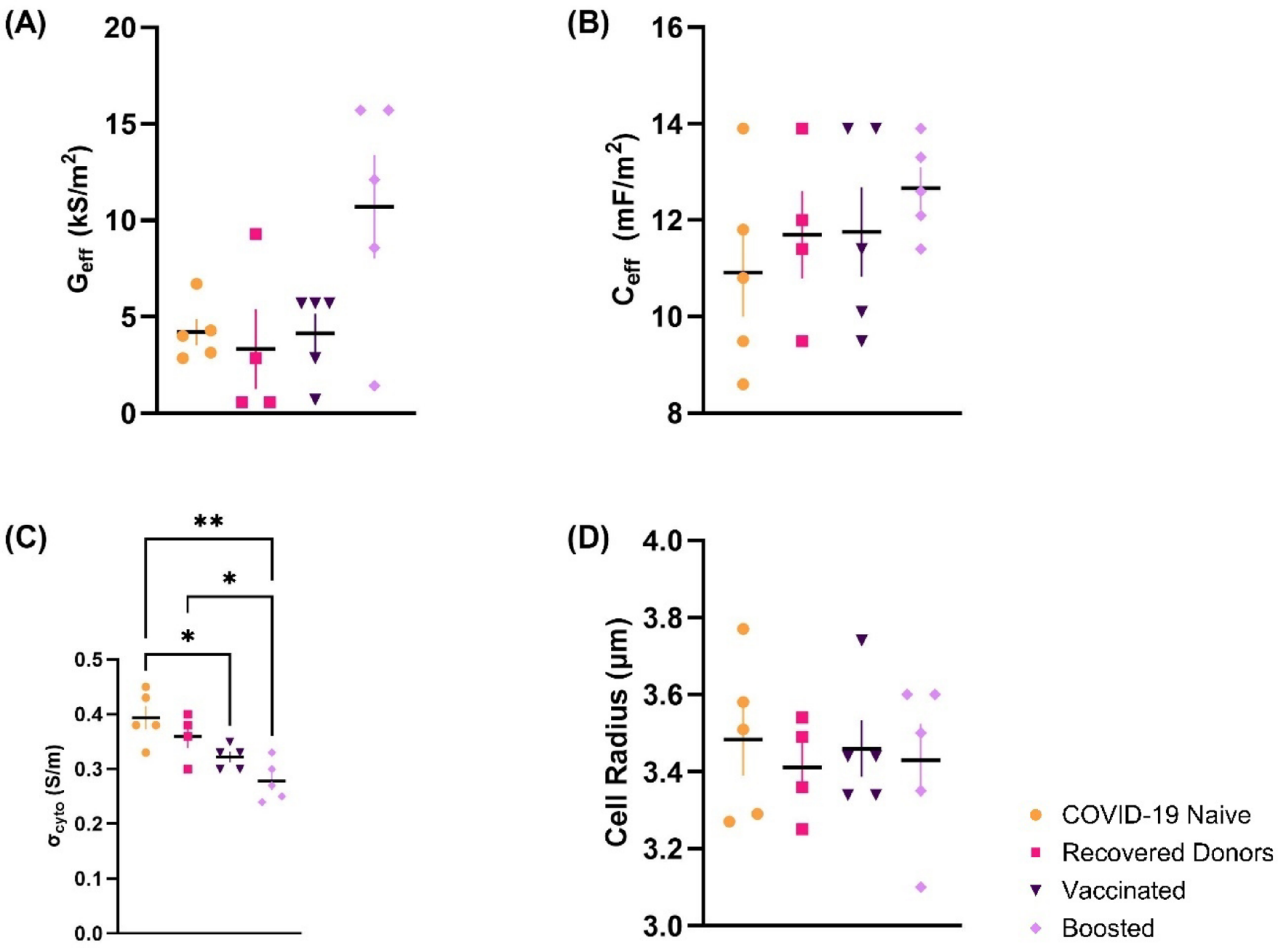
Baseline values of (**A**) G_eff_ (**B**) C_eff_ (**C**) σ_cyto_ (**D**) cell radius in unchallenged freshly isolated PBMCs from unmatched donors who are COVID-19 naïve (orange circles: n = 5), have recovered COVID-19 prior to receiving the vaccine (pink squares: n = 4), completed the two-dose SARS-CoV-2 vaccine (dark purple inverted triangle: n = 5) and received a booster dose (lilac diamonds: n = 5). Values are calculated from MATLAB-fitted models of DEP spectra which possessed an r^2^ value of over 0.8. Statistical significance calculated using one-way ANOVA followed by Tukey’s multiple comparisons (*p < 0.05). Mean lines plotted (± SEM).

The values of G_eff_, C_eff_, and σ_cyto_ were first analyzed from unchallenged PBMCs from individuals who were COVID-19 naïve (hereafter, “Naïve”), had recovered from an infection of COVID-19 prior to vaccination (“Recovered”), had received the standard two-dose SARS-CoV-2 vaccine (“Vaccinated”) or had received a third dose of SARS-CoV-2 vaccine 6–12 months after receiving the vaccine (“Boosted”). Extracted parameters of unmatched donors, plus measured radii, can be seen in Fig. 1 and Table 2. Values of G_eff_ were deemed not normally distributed, whereas C_eff_, σ_cyto_, and cell radius were found to be normally distributed. The mean value of G_eff_ (Fig. 1a) was markedly greater in unmatched donors from Boosted cohorts at 10.7 ± 2.7 kS/m^2^ than Naïve donors at 4.2 ± 0.7 kS/m^2^, but this was not statistically significant (> 0.999). There was also no statistical difference identified in baseline values of cell radius (Fig. 1d) and C_eff_ (Fig. 1b) between cohorts, although the values of C_eff_ in Boosted donors were marginally greater at 12.7 ± 0.4 mF/m^2^ than COVID-19 naïve donors at 10.9 ± 2.1 mF/m^2^. In contrast, the mean values of σ_cyto_ (Fig. 1c) decreased as the number of vaccinations increased, with a significant decrease from 0.39 ± 0.02 S/m in Naïve to 0.32 ± 0.01 S/m in Vaccinated (*p = 0.049) and 0.28 ± 0.02 S/m in Boosted donors (**p = 0.0012). There was also a significant decrease between Recovered and Boosted populations (*p = 0.032).

**Table 2:** Values of G_eff_, C_eff_, σ_cyto_ and cell radius of unchallenged PBMCs from unmatched COVID-19 naïve (n = 5), recovered COVID-19 (n = 4), second dose (n = 5) and third dose (n = 5) donors.

Donor Cohort	Geff (kS/m2)	Ceff (mF/m_2_)	σcyto (S/m)	Cell radius (µm)
COVID-19 Naïve	4.2 ± 0.7	10.9 ± 0.9	0.39 ± 0.02	3.48 ± 0.09
Recovered COVID-19	3.3 ± 2.1	11.7 ± 0.9	0.36 ± 0.02	3.41 ± 0.07
Vaccinated	4.1 ± 1.0	11.8 ± 0.9	0.32 ± 0.01	3.43 ± 0.07
Boosted	10.7 ± 2.7	12.7 ± 0.4	0.28 ± 0.02	3.41 ± 0.07

Mean values ± SEM

The increase in G_eff_ and decrease in σ_cyto_ shown in Fig. 1 suggest that there may be changes in the baseline electrophysiological properties of PBMCs following a SARS-CoV-2 vaccination, which are not observed in the recovered COVID-19 cohort. However, it is worth noting that psychological stress causes systemic inflammation^65^ and significantly higher plasma levels of cytokines such as IL-6 in individuals with higher cortisol levels^66,67^; which may be an important factor given that the Recovered cohort all acquired COVID-19 during the early part of the pandemic.

### Following COVID-19 exposure, σ_cyto_ of PBMCs is reduced over time

Given the changes in the immune system over time following natural infection and vaccination, such as increased SARS-CoV-2 IgG specific memory B-cells^33^ and decreased anti-SARS-CoV-2 T-cells^30^, it is also important to consider whether there were any differences in the time between exposure (either vaccination or natural infection with SARS-CoV-2) and measurement. Although the humoral and cellular immune response to SARS-CoV-2 has been shown to wane over time, this is estimated to occur after 6–8 months^34^ which is outside of the timeframe of these experiments.

**Fig. 2:**
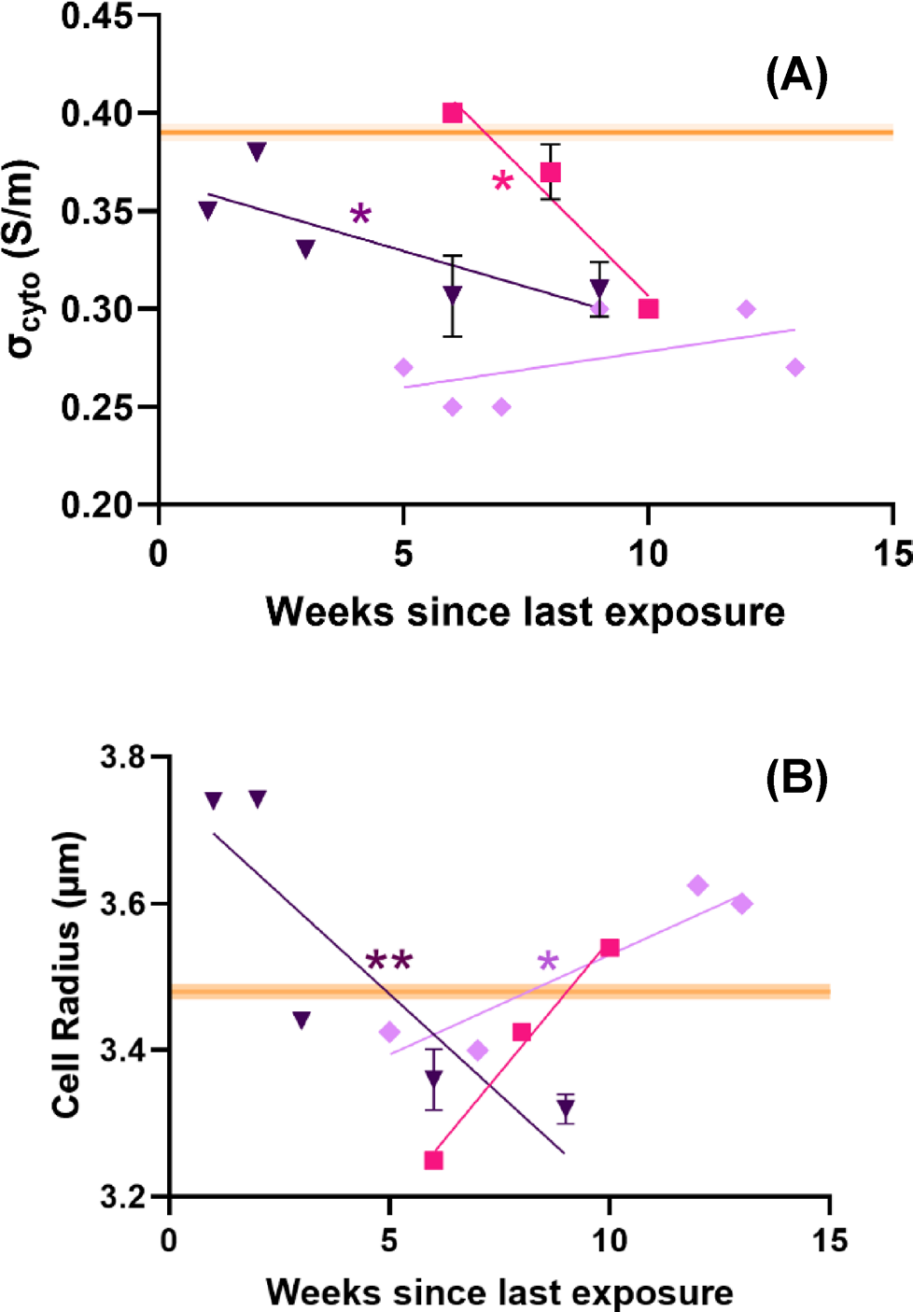
Baseline values of (A) σ_cyto_ and (B) mean cell radius against the time since exposure (weeks) to COVID-19 either through natural immunity or vaccination and the date of the experiment. Recovered COVID-19 donors (pink squares, n = 4 donors), Vaccinated donors (purple inverted triangles, n = 5 donors) and Boosted donors (lilac diamonds, n = 7 donors); best-fit lines plot simple linear regression models. Slope is significantly non-zero *p < 0.05, **p < 0.01. The orange horizontal line indicates the baseline value for Naïve donors.

We sought to probe the significant alteration of σ_cyto_ more deeply by considering whether the effect is constant or time-variant. To do this, σ_cyto_ was plotted against the weeks (at time of experiment) since the most recent of either testing positive for COVID-19, or receiving the vaccine examined for all donors from the Recovered, Vaccinated and Boosted donor cohorts (Fig. 2a). Since the Naïve population had no covid exposure, it does not have a time component and is represented here as a semitransparent orange line denoting the mean value. Intriguingly, σ_cyto_ was observed to fall from the baseline value in all cases following exposure to COVID-19 or the vaccine; both the Vaccinated and Recovered populations showed a significant trend starting at baseline and slowly moving to a terminal value of around 0.3 S/m after approximately 6 weeks (Vaccinated, *p = 0.044) to 10 weeks (Recovered, *p = 0.036). Since the Vaccinated cohort would have had a prior, initial first dose of the vaccine typically 8–19 weeks prior to the second dose used here as a benchmark, the duration from exposure to the observed change in response may be similar. When Boosted donors were analyzed, it was found that the best-fit linear regression was not statistically distinguishable from a horizontal line, suggesting a steady-state value. Interestingly, this value was the same as the terminal values for the Recovered and Vaccinated cohorts. Cell radius (Fig. 2b) was also shown to change with duration since exposure, correlating with a significant *decrease* in Vaccinated (**p = 0.0069) and a significant *increase* in Boosted (*p = 0.042). No significant trends were observed in G_eff_ or C_eff_ over time as the coefficients of the linear regression model were not statistically greater than zero (*p* > 0.05) for any donor cohort.

### Stimulation with SARS-CoV-2 S-protein RBD alters all cohort PBMCs except Naïve

**Fig. 3:**
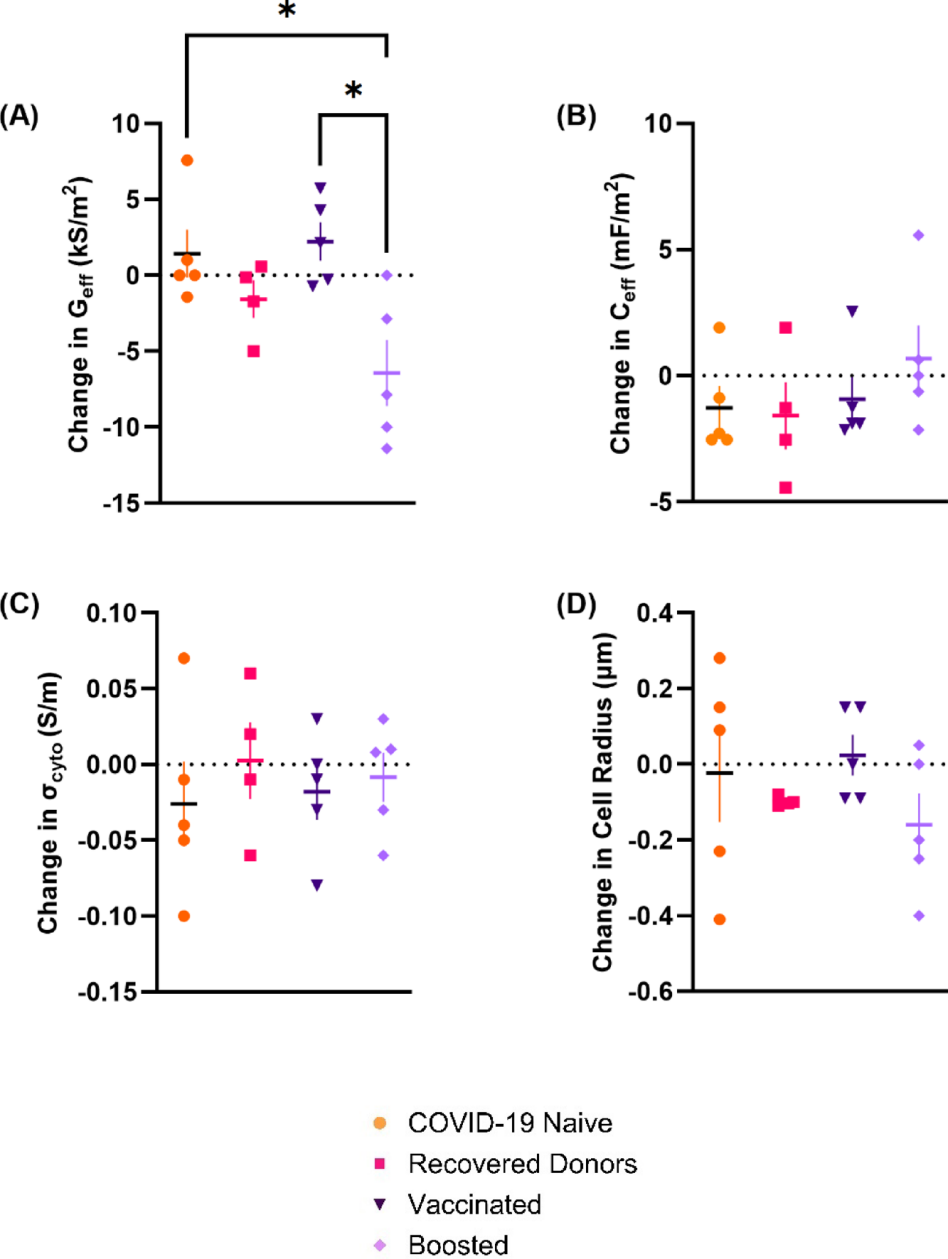
Normalized values of (**A**) G_eff_ (**B**) C_eff_ (**C**) σ_cyto_ (**D**) cell radius in 3-h SARS-CoV-2 RBD stimulated PBMCs from unmatched individuals who are COVID-19 naïve (orange circles: n = 5), have recovered COVID-19 (pink squares: n = 4), been Vaccinated by a SARS-CoV-2 vaccine (dark purple inverted triangle: n = 5) and Boosted (lilac diamonds: n = 5). Values are calculated from MATLAB-fitted models of DEP spectra which possessed an r^2^ value of over 0.8. Statistical significance calculated using a one-way ANOVA followed by Tukey’s multiple comparisons. Mean lines plotted (± SEM).

We also sought to assess whether the electrical properties of PBMCs alter when challenged by in vitro stimulation with SARS-CoV-2 S-protein RBD, both to observe changes associated with activation in an immune cell population, and to see whether this response diminished over time. To assess whether challenge altered the cells in an electrically observable manner, differences were measured in PBMCs before and after RBD-stimulation. Donor normalized changes in G_eff_, C_eff_, σ_cyto_, and cell radius are plotted in Fig. 3. Normalized values of G_eff_ were significantly different between Naïve and Boosted donors (*p = 0.023), and between Vaccinated and Boosted donors (*p = 0.012). No statistically significant differences were observed in normalized values of C_eff_, σ_cyto_, or cell radius. To probe this further, we also investigated the parameters as a function of time, but no individual parameter showed statistical significance.

To better reflect a measure of overall response, we examined the change in the mean difference value (MDV) by subtracting the MDV of the cells before challenge from the value after challenge; if the cells do not respond we expect this to be near zero, with values further away representing a greater response. The MDV is a general parameter empirically describing DEP response, has the advantage of not requiring the measurement of cell radius, and has been shown to be effective in cancer diagnosis^48,50^. As expected, the Naïve group showed little or no response to RBD stimulation, with the average difference being approximately zero and with all values within the bounds of ± 0.1. All cohorts with prior COVID-19 exposure exhibited responses which had non-zero means; whilst none were significantly different to the control group, this is to be anticipated where the responses are likely to trend towards nil response over time as immunity is lost.

**Fig. 4:**
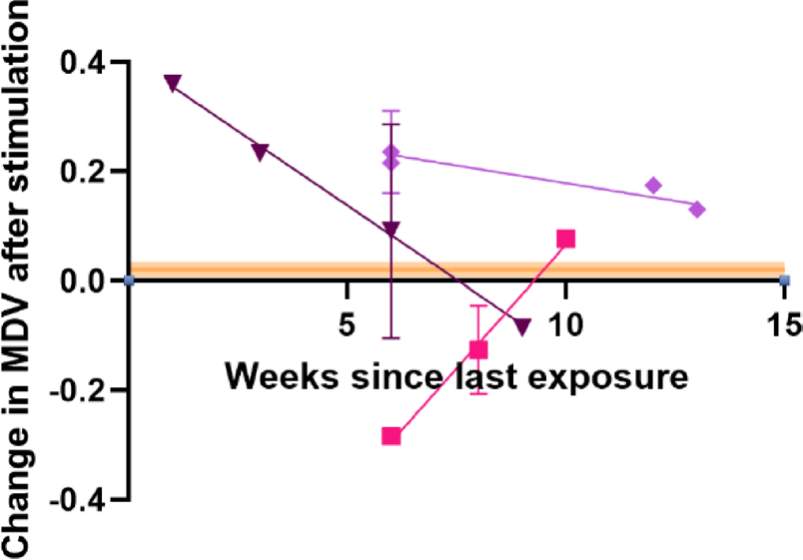
Change in MDV following 3-h RBD-stimulation in donors over time for Recovered (squares: n = 4), Vaccinated (triangles: n = 5) and Boosted (diamonds: n = 7) donors. Fitted lines plot simple linear regression models. The orange line indicates the value for the Naïve cohort. The X-axis indicates the number of weeks between the date of the experiment and the last date of COVID-exposure (either a positive test or date of last vaccination).

When we plotted the MDV as a function of time, we achieved the results shown in Fig. 4. As before, since the COVID-naïve group had no time since previous exposure, they are not represented here; instead, the orange line shows the mean value. Similarly, all cohorts showed a difference to zero at short timescales from exposure, and all exhibited a slope towards zero over time. None of the slopes achieved statistical significance, largely due to the low number of samples. However, p-values were observed close to significance, with *p* = 0.08 observed, whilst the fitted slopes for both second and third dose were *p* = 0.13. However, straight-line fits achieved r^2^ values of 0.5–0.83. Interestingly, the slopes for both Recovered and Vaccinated participants intersected the x-axis (representing no immune response) after 60 days; the response after the third dose persisted for far longer and was still producing positive responses after 3 months. First dose responses were all in the ± 0.1 band identified from the control cohort, the Vaccinated cohort dropped into this band around day 40, and the Recovered cohort around day 60. The Boosted cohort produced a weaker response which remained elevated throughout the trial but was falling toward that band around day 90. This suggests the MDV may indeed have the potential to act as a CoP for COVID-19 immunity, and by extension, for other diseases as well.

### No changes observed in gene transcription in memory B- or T-cells related after RBD-stimulation

**Fig. 5:**
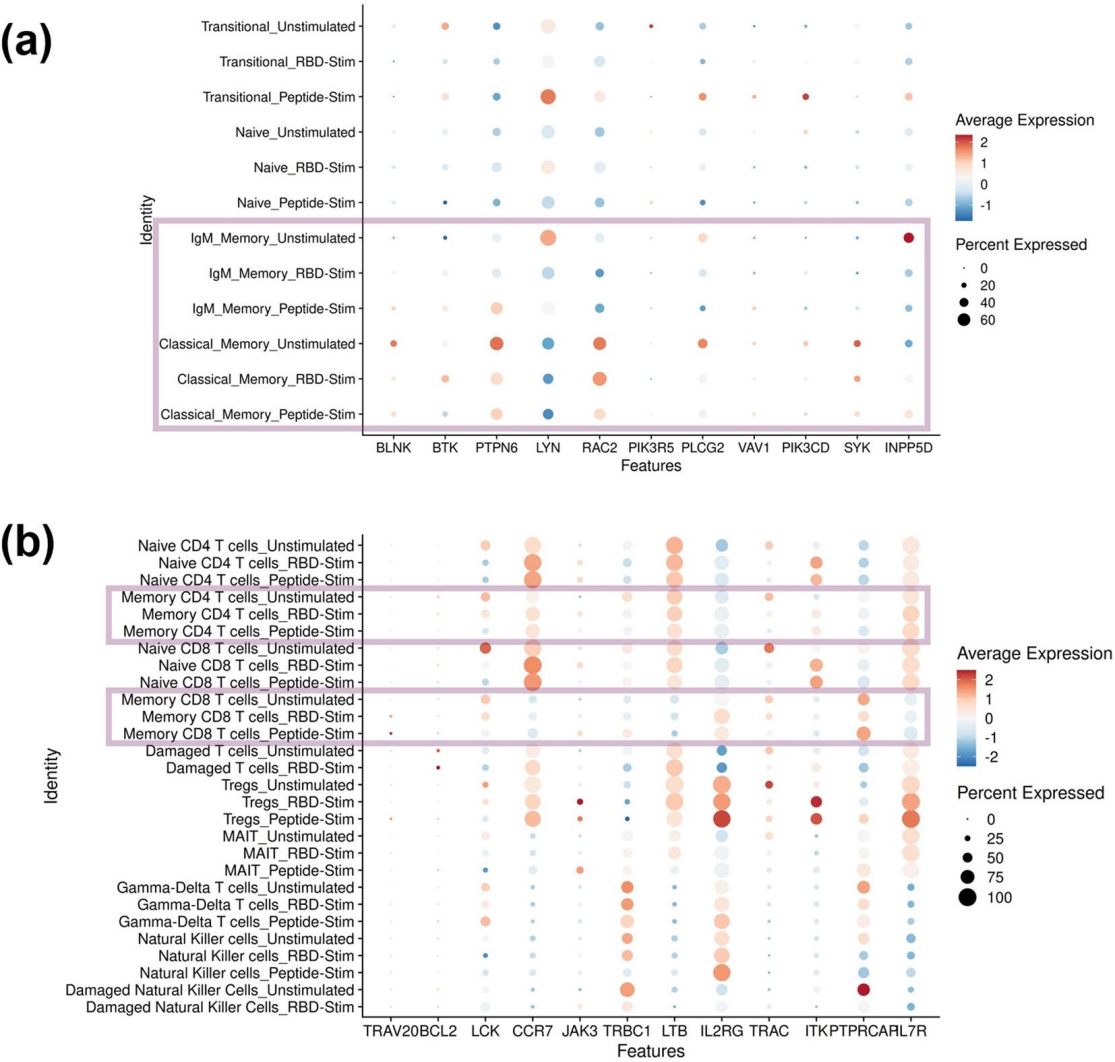
There were no significant changes to genes related to BCR or TCR signaling in adaptive memory cell populations. The average expression of (**A**) BCR or (**B**) TCR-related signaling genes were calculated for each cellular subtype. The larger the dot the higher the percentage of cells in the cluster expressing that gene and the color gradient represents the direction of regulation (red = upregulated, blue = downregulated).

**Fig. 6:**
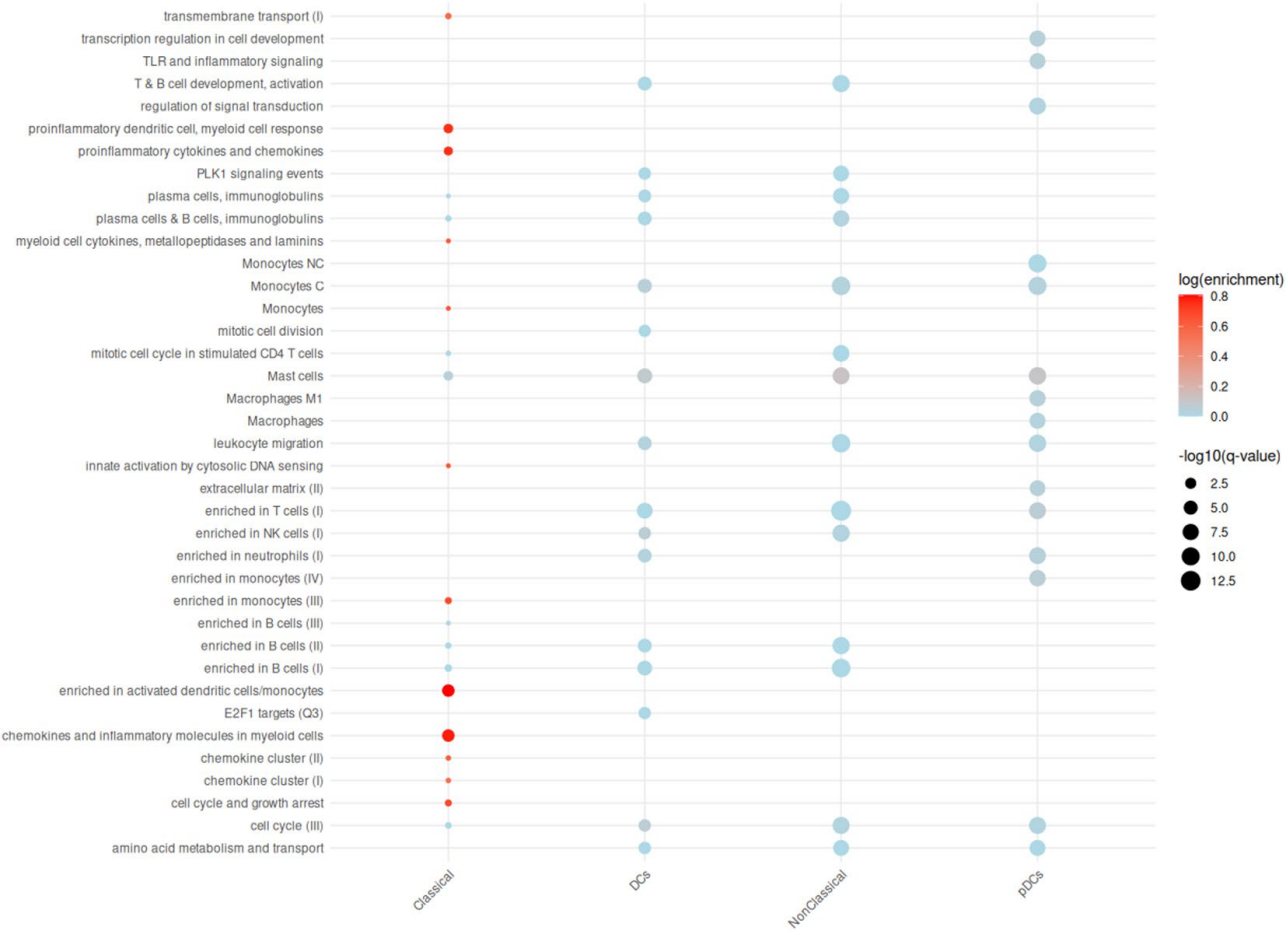
Classical Monocytes upregulate genes associated with pro-inflammatory chemokines and cytokines. Dot plot-based visualization of which pathways are up or downregulated in response to RBD stimulation when compared to unstimulated cells. Color scale represents direction of regulation, red = upregulated, blue = downregulated. The size of the dot is proportional to the *p*-value where the bigger the dot the smaller the p-value and thus more significant. MNPS = mononuclear phagocytic cells, DCs = dendritic cells, pDCs = plasmacytoid dendritic cells, Classical = classical monocytes, NonClassical = non-classical monocytes.

Analysis of the scRNAseq data identified there were no changes in gene transcription in memory B-cells or memory T-cells related to BCR or TCR signaling in response to 3-h RBD-stimulation shown in Fig. 5. Instead, cells of the innate immune system, such as classical monocytes and DCs had upregulated gene expression of pro-inflammatory chemokines and cytokines including TNF, IL1A and IL-6. scRNAseq experiments identified no changes in gene transcription in adaptive immune response cells 3-h post-RBD stimulation. Changes were observed in cells of the innate immune response (Figs. 5 and 6). Although transcriptomic changes do not always correlate with phenotypic changes, this indicates that the adaptive immune response was not activated by 3-h RBD-stimulation, but the innate immune response was.

## Discussion

This study represents the first detailed DEP-led study of immune cell response to infection. Whilst the response of individual cells has been measured following viral infection using related techniques^68^, the *systemic* response has not. Using heterogeneous PMBC populations rather than separate cell types, we were able to rapidly assess broad-spectrum response to immune challenge with the aim of testing the hypothesis that DEP could be used to assess immune response when challenged with SARS-CoV-2 spike proteins. Measurement of a CoP would have been beneficial in the early part of the pandemic, when this study began, and when the degree of protection afforded by infection had not been determined.

The majority of this study was conducted in the first half of 2021, in order to examine the potential for immune response measurement before the implementation of widespread vaccination. The Covid-naïve and Recovered populations dated from the earlier part of this period, whilst the Vaccinated populations were measured following the start of the national vaccination rollout. Consequently, the infections in the Recovered section were almost exclusively of the Alpha variant, though this was not confirmed genetically.

Concurrent with the study, during early 2021 the UK was under COVID-19 lockdown restrictions, meaning the immune systems of those in the study were unchallenged from other respiratory diseases. The reduction of interpersonal contact during lockdown meant that general respiratory infection was low among the sampled population. Conversely, some conditions during the pandemic—in particular the reducing number of Covid-naïve people, particularly once mass vaccination began, and restrictions on contact with those who had recently been infected—limited the opportunity to recruit participants additional to those presented here.

Considering the results presented, the first notable observation was that the baseline DEP response of PBMCs altered in cells exposed to SARS-CoV-2, either by infection or vaccination. This was observed in cells prior to immune challenge, suggesting an inherent change in the electrophysiological state of (or within) the PBMC population. Given the lack of immune challenge in the period preceding analysis of the Covid-Naïve population due to the lockdown, this may represent a “quiescent” state where the immune response has been inactive or some time (at this point, donors had been in lockdown for up to a year).

Following exposure, the electrophysiology of the population changed. There are two possible sources for such a change; either the individual cells that make up the PMBC population have changed their properties, or the relative numbers of different constitutive cell types within the PBMC population has changed, assuming these cells have different properties. In this work, all DEP spectra were modelled as a single population; features indicating multiple populations in the DEP spectrum^69,70^ were not evident, with the single spectrum representing the mean properties across the whole cell population^69,70^.

The electrical properties of the cell types that comprise the PBMC population have been reported in the literature. For example, Vykoukal et al.^71^ measure the DEP crossover frequencies (and by extension, C_eff_) exhibiting a range of around 50% from lowest (Eosinophils, 9.4 mF/m^2^; B-lymphocytes, 9.9 mF/m^2^) to highest (monocytes, 14.2 mF/m^2^; T-lymphocytes, 13.3 mF/m^2^). However, measured values of C_eff_ remained notably stable across the first three conditions (Fig. 1b); however, there may be a non-significant rise in C_eff_ for the Boosted population, potentially indicating a shift in composition with (per the results of Vykoukal et al.) a reduction in B-lymphocytes, or an increase in T-lymphocytes or monocytes. Similar changes have been observed in studies comparing σ_cyto_ among WBC populations. For example, the σ_cyto_ of B-lymphocytes in healthy donors was measured at 0.73 ± 0.18 S/m using electrorotation, compared with 0.56 ± 0.10 in monocytes^72^.

There is published evidence of changes in the proportions of cell types that make up the PBMC population. As the DEP spectrum is an aggregate measure of all PBMCs within the cell sample, it is possible that a change in the contribution of a subpopulation (such as T-lymphocytes) due to altered cell counts following exposure results in a different overall DEP spectrum. For example, Manunta et al.^73^ found PBMC fractions recovered using density gradient centrifugation from early COVID-19 patients had reduced circulating lymphocytes and monocytes, in addition to markedly greater numbers of low-density neutrophils with altered cell size, than healthy controls. However, no difference in average cell radius was measured between donors (Fig. 1d).

As shown in Fig. 6, RBD stimulation resulted in activation of the innate immune response. This corresponds with previous studies, which have reported the SARS-CoV-2 spike protein activates innate immune response Toll-Like Receptors (TLR), including TLR2^74^ and TLR4^75^. Our study detected electrophysiological differences in PBMCs in vaccinated and boosted donors spanning 15 weeks post-exposure. Studies have shown that SARS-CoV-2 specific CD4^+^ and CD8^+^ T-cell immune responses are established and maintained in naturally infected and vaccinated donors between 7 and 95 days post-exposure. In BNT162b2 vaccinated donors, robust expansion of fully functional SARS-CoV-2 specific CD8^+^ T-cells occurs within 1 week of vaccination^76^, and expansion of SARS-CoV-2 specific CD4^+^ T-cells occurs within 29 days of vaccination^77^. Strong IFNγ^+^ or IL-2^+^ CD8^+^ and CD4^+^ T-helper type 1 cell responses are maintained 9 weeks after booster dose^79^, and polyfunctional CD4^+^ T-cells persist 141–210 days following the second vaccination^78^. Similarly, SARS-CoV-2 specific CD4^+^ T-cells have been detected as early as day four PSO in recovered COVID-19 donors^35^ and memory T-cell responses within a few weeks PSO^39^.

Another potential reason why the electrical properties of PBMCs changed following SARS-CoV-2 exposure is that PBMCs are more activated or have a greater reactivity. Langgartner et al.^79^ reported that during ex vivo culture, PBMCs isolated from individuals who received the BNT162b1 vaccine secrete 600 times more pro-inflammatory IL-6 and two times more anti-inflammatory IL-10 in basal conditions than non-vaccinated individuals^79^. There is also growing evidence of significant changes in PBMC gene expression after vaccination, including genes involved in TNF-α signaling via NF-κβ, IL6-JAK STAT3 signaling and inflammatory responses^80^. This is supported by Bergamaschi et al.^81^ who found the first BNT162b2 vaccination causes systemic inflammation, including increases in IL-15 and IFN-γ, and increases in TNF-α and IL-6 following the second vaccination. Additionally, vaccination relies on the long-term production of NAb by long-lived plasma cells or memory cells during the germinal center (GC) reaction in a secondary lymphoid organ such as lymph nodes or the spleen^82^. As such, memory B-cell activation and differentiation in GCs following vaccination has been documented to last for months^83^, although how many of these activated memory B-cells enter peripheral circulation is unknown, or whether there would be sufficient cells to affect the congregate DEP spectrum. Following natural infection, memory T-cell responses present within a few weeks PSO^39^ and have been shown to persist, as well as memory B cell responses, for at least 3 months^33^. Moreover, levels of memory B-cells have been reported to increase in the months following vaccination^84^ and natural infection, including a prominent population of IgG^+^CD27^+^CD21^+^ RBD-specific memory B-cells which increased between 1 and 3 months^33^. However, PBMC immunophenotyping using flow cytometry is required to investigate this.

We also set out to test the hypothesis that DEP could act as a tool to measure immune response, acting as a CoP. We identified the MDV as a potential marker for this, as seen in Fig. 4. MDV has been demonstrated as a useful indicator of differential DEP response due to its not requiring measurement of cell radii, making it more amenable for rapid diagnostic testing; it has already been demonstrated as an effective diagnostic in both oral cancer^49^ and bladder cancer^50^. It also offers the benefit of combining changes in both G_eff_ and σ_cyto_. When MDV was measured following stimulation, it showed a measurable change which may be attributable to the acute changes in the cells in response to challenge. Furthermore, that change was found to diminish over time for first contact (Recovered) and first/second contact (Vaccinated) to the virus; DEP responses observed to both conditions suggest that in these cases, immune response return to baseline after approximately 2 ^1^/_2_ months, in line with contemporary estimates of protection of 3 months^85–88^. The Boosted response also showed signs of tending towards baseline but more slowly; extrapolation of the line in Fig. 4 would suggest the immune response remains present for 6 months or more. Whilst this is not definitive proof that DEP can provide a CoP, it suggests that further investigation is warranted, perhaps using a different pathogen.

Furthermore, it is possible that the decrease in σ_cyto_ may indirectly correlate with immune protection. One hypothesis to this decrease in σ_cyto_ is that the proportion of PBMCs of different subtypes continually change following vaccination, including an increase in PBMCs over time with a lower σ_cyto_. Recent work^89^ has shown that σ_cyto_ acts as a correlate of the plasma membrane potential V_m_, as does G_eff_^90^. V_m_ is known to play a number of roles in immune cells, including depolarization being a key part of T-lymphocyte activation^91,92^ and macrophages^93^.

The study also highlighted differences in the PBMC response to natural infection vs. vaccination. Intriguingly, the Recovered response as measured by MDV was shown to act in the opposite direction to those following vaccination and boosting, with the MDV dropping following stimulation in the former case and rising in the latter two, before returning to baseline over time. Similarly, the radius of Vaccinated cells was found to decrease shortly after exposure, whereas for Boosted and Recovered donors it increased. As described above, there are two potential reasons for changes in value; changes in cell subset concentration, and change in the physical properties of those subsets. These different behaviors suggest that both may be present to different degrees and act in different directions. This suggests that further study into the electrophysiology of PBMC subpopulations in future work may reveal new insights about immune function, and in particular on potential differences between immune response and the source of exposure, by vaccination or infection.

## Conclusion

As the COVID-19 pandemic demonstrated, there can be sudden, unexpected and unprecedented requirements for methods to rapidly understand a new pathogen and the body’s response to it, in order to both understand its pathogenicity and to develop and assess tools against it. In this paper we have demonstrated the potential DEP offers to act as a non-molecular CoP. Since the technique measures the immune response to challenge, it is potentially applicable to assessing the immune response to any challenge, and hence can be rapidly adapted to new pathogens as they arrive. It also offers a method for assessment of the efficacy and period of protection. Further work with other pathogens will be required to determine the general applicability of the method in wider immunology.

## Electronic supplementary material

Below is the link to the electronic supplementary material.

Supplementary Material 1

## Data Availability

Dielectrophoresis data are available from the corresponding authors on request. The gene expression data is available from Array Express, Accession number: E-MTAB-14606.
