# Supplementary material for: Changes in peripheral blood mononuclear cell electrical properties in response to viral exposure and vaccination
Source: Sci Rep. 2025 Jul 9;15:24583. doi: 10.1038/s41598-025-08724-6 (PMC12238384; doi:10.1038/s41598-025-08724-6)
Supplement: Supplementary file 1 — Supplementary Material 1 [file 41598_2025_8724_MOESM1_ESM.docx]

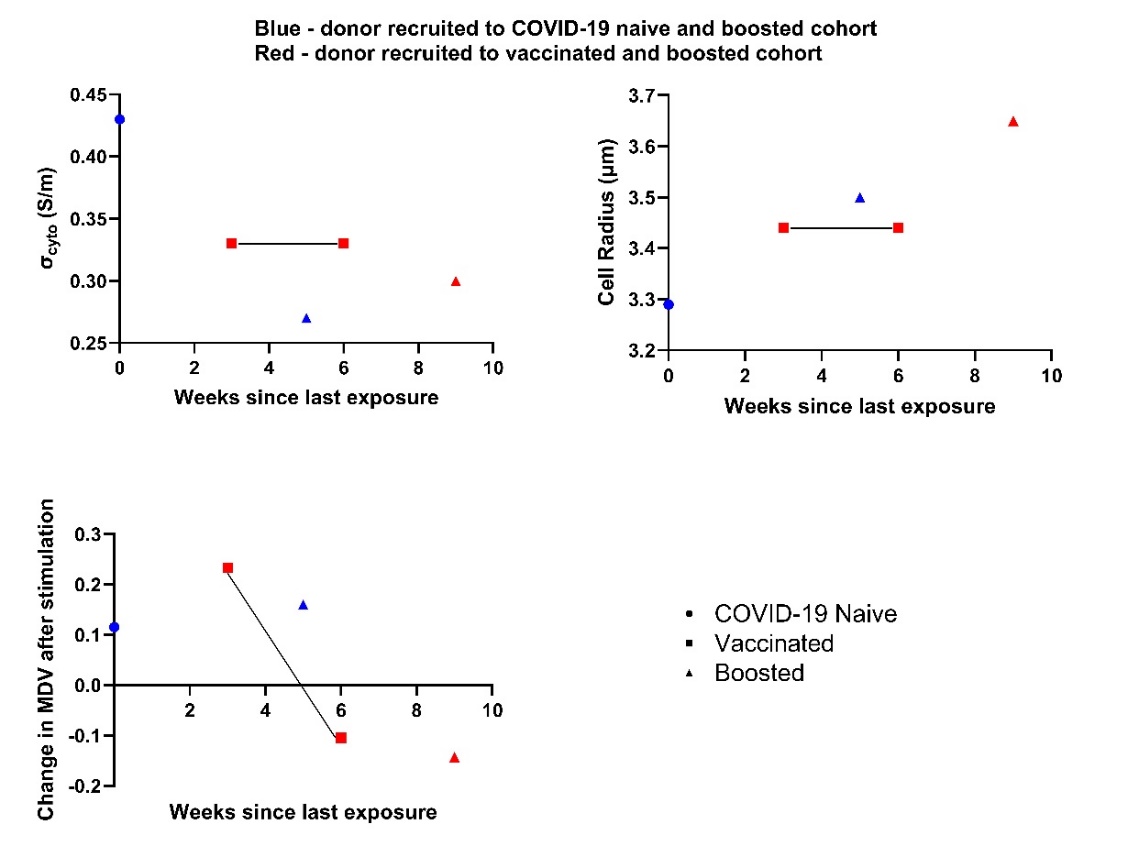


**Supplementary Figure 1.** σ_cyto_, cell radius and change in MDV for two donors who appeared in multiple cohorts.


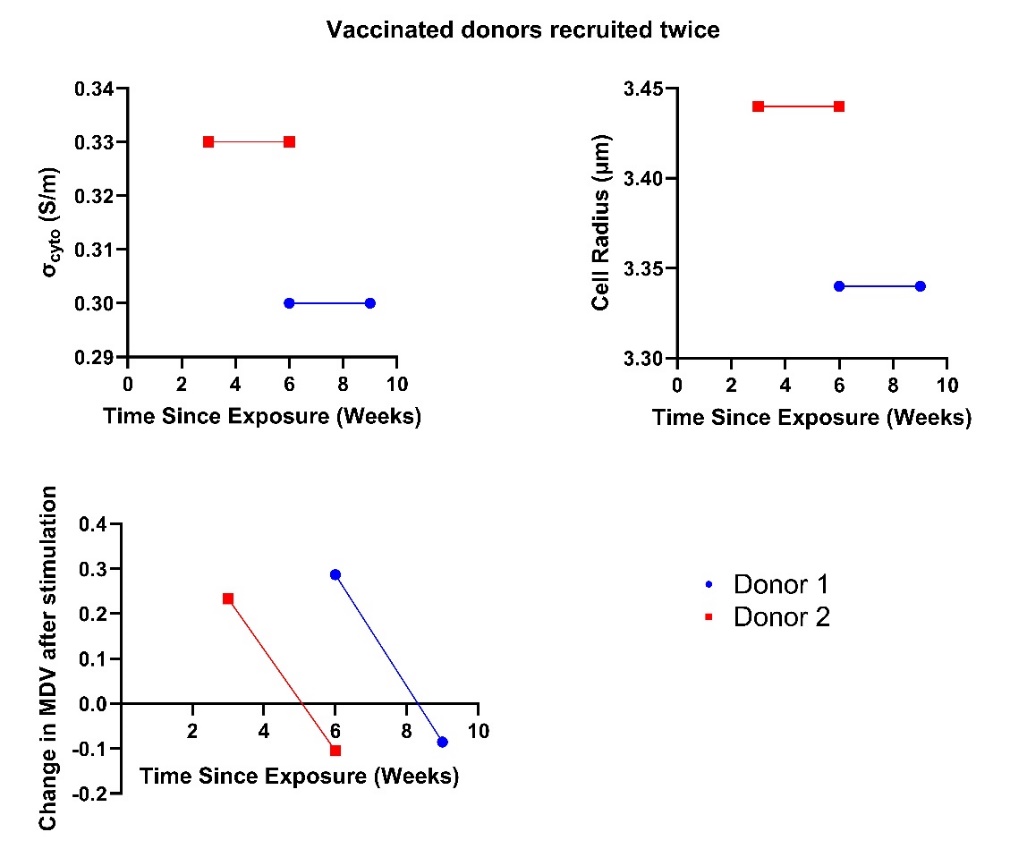


**Supplementary Figure 2.** σ_cyto_, cell radius and change in MDV for vaccinated donors who were analysed twice at different times after vaccination.
